# How might we motivate uptake of the Dual Prevention Pill? Findings from human-centered design research with potential end users, male partners, and healthcare providers

**DOI:** 10.3389/frph.2023.1254953

**Published:** 2023-11-01

**Authors:** Wawira Nyagah, Kate Segal, Jess Feltham, Alex Ash, Jocelyn Major, Moowa Masani

**Affiliations:** ^1^ AVAC, Product Introduction and Access, New York, NY, United States; ^2^ M&C Saatchi World Services, London, United Kingdom; ^3^ REACH Consumer Insights, Cape Town, South Africa

**Keywords:** pre-exposure prophylaxis, oral contraception, HIV prevention, family planning, multi-purpose prevention technologies, end users, human-centered design, demand generation

## Abstract

**Introduction:**

Multipurpose prevention technologies (MPTs) combining contraception with HIV prevention offer a promising solution to uptake and adherence challenges faced with oral pre-exposure prophylaxis (PrEP). The Dual Prevention Pill (DPP), which combines oral PrEP with an oral contraceptive pill (OCP), could address unmet need for family planning (FP) and HIV prevention. This study aimed to identify barriers and motivators for DPP uptake to inform the development of a DPP demand generation strategy and broader introduction efforts for MPTs.

**Materials and methods:**

Qualitative, ethnographic research employing human-centered design techniques was conducted in Kenya, South Africa, and Zimbabwe. A research consortium conducted 45 immersions, 34 key informant interviews, and 12 friendship circles with potential end users, male romantic partners, healthcare providers (HCPs), and cultural commentators. Creative concepts were subsequently co-created and validated in workshops with end users, male partners, and HCPs.

**Results:**

Four major themes emerged. Women struggled to balance personal motivations with societal expectations. Relationship goals strongly influenced sexual and reproductive health decisions, particularly related to financial security and social status. Negative experiences, such as untrustworthy partners, were significant triggers for OCP and PrEP use. Lastly, male partners were concerned about the DPP upending gender norms but held more positive individual attitudes. Five initial audience segments for the DPP were identified: women seeking enjoyment outside of their primary relationship; new mothers adhering to social norms; women wanting to maintain romantic relationships; women at risk of unintended pregnancy; and women with unfaithful partners. Segments informed the development of three communication themes, with the preferred route highlighting the DPP as a tool to prepare for life's unpredictability.

**Discussion:**

To effectively generate demand for the DPP, several strategies should be considered. Connecting with women's diverse identities and goals and celebrating their individuality is crucial. Linking the DPP to relationship goals reframes it as a means to protect relationships rather than a risk. Leveraging negative triggers through targeted media campaigns empowers women to take control of their sexual health during challenging moments. A balance in channel placement is necessary to raise public awareness while using more discrete channels for potentially controversial messages with male partners and wider communities.

## 1. Introduction

HIV and unintended pregnancies continue to pose significant challenges for women of reproductive age, particularly in sub-Saharan Africa, with a notable unmet need for effective prevention options. In 2021, women and girls accounted for 63% of all new HIV infections, with 82% of these infections occurring among adolescent girls and young women (AGYW) aged 15–24 (1). Furthermore, there is a persistent challenge of unmet need for family planning among women of reproductive age in sub-Saharan Africa, with an estimated 24.2% of married or in-union women having an unmet need for modern contraception (2). Among AGYW globally, younger AGYW (15–19 years old) exhibit higher levels of unmet need compared to older AGYW (20–24 years old) (3).

Over the past decade, several biomedical HIV prevention modalities have become available, including tenofovir-based oral pre-exposure prophylaxis (PrEP), the dapivirine vaginal ring (PrEP ring), and injectable cabotegravir (CAB for PrEP). Studies such as the TRIO (Tablets, Ring, Injections as Options) study in Kenya and South Africa and the CUPID study in Uganda and Zimbabwe have found that participants would prefer a combined product for HIV and pregnancy prevention over separate products (4, 5). Discrete choice experiments have also shown higher demand for multipurpose prevention technologies (MPTs) among women interested in HIV prevention compared to single-indication products (6).

**Figure 1:**
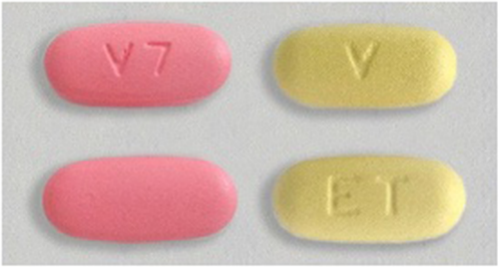
First-generation DPP tablets.

MPTs that combine contraception and HIV prevention have the potential to address the challenges of uptake, adherence, and societal stigma seen with oral PrEP (7, 8). The Dual Prevention Pill (DPP), which combines oral PrEP and an oral contraceptive pill (OCP) in a single, co-formulated, daily pill, is the MPT closest to market. The first-generation DPP will have a 28-day regimen with 21 combination PrEP/OCP tablets and 7 PrEP-only tablets, which maintain protection against HIV while allowing for monthly bleeding (Figure 1). The DPP, as the first MPT containing PrEP, could help address the unmet need for family planning and increase oral PrEP use, thereby reducing the risk of HIV infection (9). Moreover, the development and rollout of the DPP will lay the groundwork for future MPT options in the research pipeline (10). As the number of biomedical PrEP modalities increases, it is critical to understand the individual-level drivers and barriers to PrEP uptake, as well as the factors influencing decision-making in this context (11). Lessons from past demand generation efforts for HIV prevention and family planning have shown that increasing awareness and understanding of new product options alone is not sufficient to drive uptake and sustained use. Additional strategies are required to address barriers and shape attitudes towards prevention. Demand creation for PrEP should recognize the individual, interpersonal, sociocultural, and structural factors that contribute to decision-making on sexual health, and be grounded in a socio-ecological model that considers end users and their relationships to people, organizations, and their community (12). Lessons from oral PrEP rollout highlight the need to create appealing and easy-to-use products tailored to people's stated concerns and desires and to build a supportive environment in which users can make decisions (13). Engaging key influencers, such as male partners, families, peers, community, and healthcare providers (HCPs), is critical to promoting HIV prevention products for women and girls (14). Finally, empowering communities through participatory approaches and involving them in the design and implementation of demand generation strategies can improve acceptability and uptake of sexual and reproductive health (SRH) products and services (15). A successful demand generation strategy for the DPP will employ a layered approach to increase general awareness, mobilize communities, and provide support to clients and HCPs at the point of care. Employing successful approaches that include research, audience segementation, and human-centered design (HCD) can ensure that demand generation for the DPP is evidence-based, contextually relevant, and responsive to the diverse needs of the target population.

## 2. Materials and methods

### 2.1. Primary research

#### 2.1.1. Research design

AVAC and M&C Saatchi World Services led a consortium to undertake qualitative HCD research in Kenya, South Africa, and Zimbabwe from April to July 2022 to uncover potential barriers and motivators to uptake of the DPP. To inform the design of primary research, the study team first conducted a desk review of available literature to surface findings and evidence gaps. The desk review included 25 articles of 750 screened, a quantitative re-analysis of DHS datasets, and an analysis of social media conversations on key terms including birth control, contraceptives, and relationships in the three key markets using Brandwatch (16), to understand the following questions:

1. What are the values, day-to-day lives, and lifestyles of end users, and how might these be leveraged to encourage uptake of the DPP?
2. What are the opportunities and occasions in end users’ lives where the DPP brand can drive relevance?
3. Where are end users having conversations about SRH and which channels will penetrate in these locations?
4. What is the end user’s network of influence and how does this network influence decisions around sex, relationships, and SRH?
5. Which products do current OCP and oral PrEP users access, purchase, or use alongside OCP and/or oral PrEP, and what opportunities could these afford for DPP marketing?

Subsequently, the primary research aimed to fill gaps and deepen the field's understanding of the key learning questions. Participants were recruited using convenience sampling based on target audience profiles, leveraging venues in selected localities where these audiences were likely to convene within the context of study parameters (e.g., healthcare facilities, community centers). The recruitment sample included women aged 20–40 who were current OCP users, current oral PrEP users, and non-users of OCP, oral PrEP, and long-acting reversible contraceptives (LARCs). Men recruited were all in a current relationship with a woman and reflected a mix of attitudes toward the acceptability of PrEP and contraceptive use by female partners. HCPs recruited were currently providing contraception and/or HIV prevention services and represented a cross-section of cadres, including doctors, nurses, pharmacists, and providers working for non-governmental organizations (NGOs). Cultural commentators were selected for their ability to provide insights on trends, nuances, and idiosyncrasies that define how sex, relationships, and SRH are conceptualized and discussed in their local cultural contexts.

One urban and one rural location was selected in each country. Regions and provinces were shortlisted based on where the Total Addressable Market for Contraception (TAMC) for women ages 15–49 constituted a relatively large proportion of the population, and had a relatively high HIV prevalence rate for women ages 15–49 (17). These locations were reviewed by the study team to identify preferred regions and provinces, after which local field research teams recommended specific districts to conduct the research based on whether the area was a DREAMS district (PEPFAR-funded public-private partnership aimed at reducing rates of HIV among AGYW in the highest HIV burden countries) and other logistical factors.

Research was guided by an ethnographic approach to build trust and understanding between researchers and participants through repeat engagements in the field and the pursuit of a holistic understanding of participants within their cultural contexts. Because implicit motivational drivers of decision-making are not always conscious (18), a research approach that aimed to draw out responses deeper than surface level was preferred. The ethnographic process was conducted in two 3–4-hour long sessions with each participant, which provided participants space to reflect on their responses without the time pressures often associated with a single-session research process. Because research participants often struggle to have open conversations about sex, relationships, and SRH, owing to the oftentimes sensitive and private nature of these subjects, within each session, researchers employed a variety of HCD exercises designed to facilitate these discussions and ensure that all insights were grounded in an empathetic understanding of the lived realities of participants. Institutional Review Board (IRB) approval was obtained in Kenya, South Africa, and Zimbabwe. The ethnographic participant then became the lead participant in the friendship circles, comprising friends with whom the lead participant was comfortable engaging on SRH conversations. This provided an additional layer of emotional and psychological comfort during the potentially sensitive conversation.

#### 2.1.2. Data collection

A total of 45 ethnographic immersions, 34 key informant interviews (KIIs), and 12 friendship circles were conducted across the three countries (Table 1). In each country, ethnographic immersions were conducted with 13 potential end users and two male romantic partners; intimate group discussions were held with two male and two female friendship circles; and KIIs were conducted with eight HCPs. In addition, KIIs were conducted with three cultural commentators in Kenya and South Africa, respectively, and four in Zimbabwe.

**Table 1:** Primary research sample and methods per country.

Research method		Immersion (2× sessions per participant)				Friendship circle (Participant + friends)		Key informant interview	
Country	District	Current OCP users	Current PrEP users	Non-users of OCP/PrEP	Male romantic partners	Female circle	Male circle	HCPs	Cultural commentators
Kenya	Nairobi (Urban)	3	2	2	1	1	1	4	3
	Areas around Kisumu (Rural)	3	2	1	1	1	1	4	
South Africa	Johannesburg (Urban)	3	2	2	1	1	1	4	3
	Districts in KwaZulu-Natal (Rural)	3	2	1	1	1	1	4	
Zimbabwe	Harare (Urban)	3	2	2	1	1	1	4	4
	Areas around Bubi (Rural)	3	2	1	1	1	1	4	
Total participants		18	12	9	6	6	6	24	10

Fieldwork was conducted by 1–2 lead ethnographers in each country. Three HCD research tools were employed:

• **Social network mapping** was used to investigate who male and female immersion participants would seek advice from (and who they would avoid) in different scenarios, in order to understand who influences them and their level of trust in different people in their networks. Participants were invited to arrange cards representing different people in their social network on a map of concentric circles. Proximity to the center of the map correlated with how trusted that person was in a given scenario: the closer to the center, the more trusted and likely that the participant would be to seek advice from them.
• **Journey mapping** was used to understand how participants came to use oral PrEP and/or OCP and their experiences using the product. The exercise explored triggers that led participants to consider and ultimately decide to use the product; experiences at different points on their journey; sources of information (people, HCPs, and media) used throughout their journey; and the places visited at different points in their journey. Participants were provided with a journey template and a set of prompt cards to populate the template. These prompt cards required the researcher (or the participant, depending on literacy level) to write descriptions on the cards of what happened and how they felt at each stage of the journey and arrange them in a visual representation on the map.
• **Mini-Me** was used to explore the personal values and relationship goals of male and female participants. Participants were provided with a template on which to draw and annotate themselves (their ‘mini-me’) and their partner. Researchers asked questions to encourage participants to capture their personal values, aspirations, roles, and responsibilities, as well as the values and roles they wished for their ideal partner.

All ethnographic sessions were recorded with digital audio recorders and transcribed into English where relevant, with appropriate permissions. Photographs were taken to capture visual data relevant to the research with permission from participants, including the final outputs from each HCD exercise. In addition, local researchers kept field notes that recorded their key findings and insights. KIIs were conducted in English using semi-structured discussion guides designed for HCPs and cultural commentators, respectively.

#### 2.1.3. Data analysis

To maximize opportunities for the analysis process and benefit from the expertise and experience of field researchers, a series of data download sessions were convened. These sessions, led by the lead researcher, were attended by field researchers in each country and members of the study team. Three download sessions were conducted per country: one following initial pilot immersions, one mid-way through fieldwork, and one once all fieldwork was completed. During each session, field researchers provided an overview of the key findings emerging from the research. These findings were interrogated in an open discussion to identify implications for the rollout of the DPP, as well as considerations and recommendations for demand generation and marketing strategies. Each session lasted 2–3 hours; and was conducted via Zoom. Where permission was granted, photos were shared with the study team for further analysis.

Field researchers documented the final outputs from each HCD exercise through photographs. Responses to the Social Network Mapping exercises were transcribed to identify patterns in positioning of different influencers based on each scenario. HCD journeys were sorted based on their trigger points, and common themes were analyzed from the results. Values expressed in the Mini-Me responses were manually coded and analyzed.

#### 2.1.4. Development of user personas and journeys

Based on the primary research findings, a total of five user journeys and personas were created. The study team initiated a rapid analysis of the values and aspirations of male and female immersion participants to gain insights into their key motivators and values. This analysis aimed to explore how these factors intersected with their SRH goals. The data utilized for this analysis included information derived from the ‘Mini-Me’ exercises and conversations with participants regarding their personal values.

Subsequently, the study team examined the barriers to the uptake and sustained use of SRH products that were reported at different stages of the research process. These barriers were identified through a combination of methods, including a desk review, discussions with male and female participants, HCPs, and cultural commentators, as well as social network mapping and user journey exercises.

To categorize the user journeys effectively, trigger moments for product entry were considered (Table 2). Additionally, a user journey matrix was developed to ensure that the final set of journeys encompassed the diverse data collected across nine dimensions (Table 3). Finally, each user journey was associated with a persona. The personas were created to highlight the most prevalent entry points, drivers, and barriers within specific categories. These personas provide valuable insights that can be leveraged in demand generation communications.

**Table 2:** User journey sorting.

PERSONA		VICKY (New mother - planned pregnancy)	THANDIWE (New mother - unintended pregnancy)	FAITH (Untrustworthy partner)	LINDIWE (Maintaining relationship)	ELSIE (Seeking enjoyment outside of relationship)
Total (*n* = 28)		7	2	12	4	3
Product	OCP (*n* = 15)	7	2	1	3	2
	Oral PrEP (*n* = 13)	0	0	11	1	1
Country	KE (*n* = 10)	2	1	4	1	2
	SA (*n* = 9)	1	1	3	3	1
	ZIM (*n* = 9)	4	0	5	0	0
Location	Urban (*n* = 13)	3	1	5	3	1
	Rural (*n* = 15)	4	1	7	1	2

**Table 3:** User journey matrix.

Matrix dimension	PERSONAS				
	Vicky	Thandiwe	Faith	Lindiwe	Elsie
Trigger category	New mother - planned pregnancy	New mother - unintended pregnancy	Untrustworthy partner	Maintaining relationship	Seeking enjoyment outside of relationship
Product used	OCP	OCP	PrEP	PrEP	PrEP + OCP
Awareness of options at trigger moment	High	Low	Low	Low	High for OCP, low for PrEP
Primary influence on preferred product	Partner, HCP	Older female relatives	HCP	Sister, HCP	Close friend
Online research conducted?	Yes - to form initial preferences	No	No	Yes - to check what partner's medication is for	Yes - to look up how to manage side effects
Partner aware of product usage?	Actively consulted	No	No	Informed but not consulted	No
Side effects experience	Severe	Low	Moderate	No	No
Product switch?	Yes	Yes	No	No	No
Other factors	Obtain OCP from source other than clinic		Husband disapproves of condoms	Believes condoms reduce pleasure	

### 2.2. Co-creation and validation

Based on insights obtained from the desk review and primary research, the study team took three creative routes for DPP demand generation into the field beginning in September 2022, with the aim of refining and contextualizing the creative routes for localized appeal and relevance. In Kenya, South Africa, and Zimbabwe, respectively, seven co-creation workshops were conducted with current OCP/PrEP end users (×2), end users with an unmet need for HIV prevention/family planning (×2), male romantic partners (×2), and HCPs (×1). In each market, four workshops were held in urban locations and three workshops were held in rural locations (Table 4).

**Table 4:** Co-creation workshops recruitment sample.

Country	District	Current OCP/PrEP users	Users with unmet FP and/or HIV prevention need	Male romantic partners	HCPs
Kenya	Nairobi (Urban)	4	4	4	8
	Kisumu (Rural)	4	4	4	0
Kenya total		8	8	8	8
South Africa	Johannesburg (Urban)	4	4	4	8
	KwaZulu-Natal (Rural)	4	4	4	0
South Africa total		8	8	8	8
Zimbabwe	Harare (Urban)	4	4	4	8
	Bubi (Rural)	4	4	4	0
Zimbabwe total		8	8	8	8
Total participants		24	24	24	24

During the co-creation workshops, the three creative approaches were tested using visual identities that would be incorporated into various communication materials. Elements tested included campaign taglines and creative concepts (messaging and imagery) adaptable to different audiences. The results from the co-creation workshops helped narrow down the focus to a single creative approach for the DPP.

Following the co-creation fieldwork, revised creative stimulus was developed for the selected route, building out a range of scenarios, positioning, imagery, and messaging. The subsequent validation workshops comprised four sessions in each market: two sessions with end users (current OCP/PrEP users and non-OCP/PrEP users) in one urban and one rural location; one session with male romantic partners in an urban location; and one session with HCPs in an urban location (Table 5). This validation phase was convened to assess the likeability, relevance, persuasion, acceptance, believability, comprehension, and attractiveness of the stimuli. Workshop participants first completed individual questionnaires on stimulus preferences, then participated in a collective group discussion. HCPs had shorter sessions due to fewer executions but were prompted to discuss potential tools for increasing clients’ adherence to a daily pill. The study team then refined the stimulus based on findings from validation workshops.

**Table 5:** Validation workshops recruitment sample.

Country	District	Current OCP/PrEP users and users with unmet FP and/or HIV prevention need	Male romantic partners	HCPs
Kenya	Nairobi (Urban)	8	8	8
	Kisumu (Rural)	8	0	0
Kenya total		16	8	8
South Africa	Johannesburg (Urban)	8	8	8
	KwaZulu-Natal (Rural)	8	0	0
South Africa total		16	8	8
Zimbabwe	Harare (Urban)	8	8	8
	Bubi (Rural)	8	0	0
Zimbabwe total		16	8	8
Total participants		48	24	24

## 3. Results

### 3.1. Primary research findings

Across audiences, four major insights emerged, which have significant implications for the DPP demand generation strategy.

• When considering the hypothetical use of the DPP, women often find themselves grappling with different aspects of their identity and conflicting values. (Women were not offered the DPP to use as part of this research.) Societal stigma related to both PrEP and OCP meant that the DPP was frequently perceived as in conflict with societal expectations, limiting potential uptake even when the DPP was seen to support personal motivations.
• Relationship goals exert a stronger influence on SRH decisions than health risks, particularly when they are linked to financial security and social status. Women prioritize maintaining stable, secure, and loving relationships with their partners and upholding family values.
• Male romantic partners feel the DPP could threaten traditional gender norms, but hold more positive individual attitudes. While the prevailing view was that men would not be in favor of their own partners using the DPP, male romantic partners expressed generally supportive attitudes towards the DPP, saying that it was an innovation that could help address endemic issues of unintended pregnancy and HIV in their countries and communities.
• Triggers for OCP and PrEP use for women were largely negative. Instances of partner infidelity, discovery of a partner’s secret HIV status, and other forms of untrustworthiness were significant factors motivating women to start using PrEP or OCP, and they strongly identified with the need to tackle risks beyond their control.

#### 3.1.1. Women navigate a plurality of values and identities

Research found that male and female participants were driven by complex, diverse, and sometimes opposing sets of self-focused and community-focused values. Female participants, in particular, made decisions about SRH considering multiple values simultaneously, carefully weighing the benefits and risks of various SRH options in relation to their diverse goals.

Self-focused values revolve around personal development, achievements, pleasure, excitement, wealth, and prestige of the self. The most commonly expressed self-focused values by participants were (1) *empowerment values*, characterized by the pursuit of professional success and accompanying financial security, and (2) *enjoyment values*, encompassed by sexual pleasure, new experiences, and indulgence in “the finer things” in life. Female participants who highly valued empowerment were more likely to adopt prevention products like the DPP, seeing them as a means to protect their goals from being disrupted by HIV or unintended pregnancy. Younger participants were motivated by career and educational goals, while male participants tended to prioritize pleasure and enjoyment in their personal values and daily practices.

Across all three countries, both men and women emphasized the desire to comply with societal expectations through two community-focused values. (1) *Religious and traditional values* were especially important for Kenyan and Zimbabwean female participants, who underscored the importance of women dressing respectably and modestly, as well as rural male and female participants, who highly valued being prayerful, devout, and humble. Demonstrating (2) *social status and respectability* was important for women, who wanted to be seen as accomplished, well-groomed, fashionable, monogamous, married with children, and having a happy, successful husband. In all three countries, research participants commonly associated marriage with social status and viewed it as a marker of success for women. The performance of respectability led women to conceal their usage of SRH products out of fear of stigma from the community, and only discussed SRH with extremely close confidants. Given the importance of discretion to their public image, women often reported a preference for SRH products that could be used covertly, and viewed the DPP as offering a solution to social risks associated with condoms and oral PrEP.

Women reported performing a plurality of identities depending on the social context, such as presenting themselves as more respectable with family and in-laws, while adopting a more outgoing, pleasure-seeking identity among close friends. Female participants in South Africa exhibited greater evidence of enjoyment values, including sexual pleasure, and ascribed greater value to the social status associated with being in a successful relationship. In Kenya, the desire for social respectability was linked to the fear of moral sanctions, and women sought “empowerment in secret,” such as achieving equality in sexual relations. This was specifically linked to the awareness that a high proportion of male partners were unfaithful, and a desire to “get even” and find their own enjoyment outside of marriage or their primary relationship. In Zimbabwe, adherence to religious and traditional values took precedence for participants. Whereas male participants generally split their time between work and relaxation, female participants’ daily lives consisted predominantly of either working for income or taking care of domestic and family duties, with less opportunity for indulging in leisure activities.

#### 3.1.2. Preserving romantic relationships is central to decision-making on SRH

The research found that women often depend on their relationships with male partners to fulfill various values that are important to them, particularly (1) *relationship goals*, which emphasize maintaining a stable, secure, and loving romantic relationship, and (2) *family values*, typified by family security, caring for children, and upholding a positive self-image and relationship with siblings and parents. As a result, women may be disinclined to discuss SRH issues with their partners or engage in practices that may upset them, fearing the potential loss of the benefits provided by the relationship. Female participants described their ideal partner as someone who serves as their personal protector, financial provider, and someone who will give them access to luxuries in life. These descriptions depict how women often depend on male partners to meet their basic security needs, and their desires for wealth and enjoyment. At times, participants and field researchers also highlighted the transactional nature of romantic relationships.

Participants tended to weigh “short-term” risks more heavily than “long-term” risks, for instance, prioritizing a threat to their relationship due to its perceived immediacy over health risks that are seen as longer-term and easier to discount. Preferences for SRH products were often based on which posed the least risks to their relationships. Some female participants reported agreeing to not use a condom because their male partner did not want to, even when the female participant knew the implications of this decision. Women reported keeping usage of PrEP and OCP secret from a partner or family member, some using a product only when required in order to avoid chances of detection. Decisions on SRH product use carried implications about fidelity, with one female participant in Kenya noting: “*According to him, condoms should be used by those who do not trust each other and those that go outside [of the relationship]*.”

The practice of concealing SRH product use from male partners was observed among female OCP and PrEP users, and was especially widespread among PrEP users. Women commonly stored SRH products alongside beauty products and medicines in their homes, and decanted pills into a container of their own choosing, rather than leave them in the original packaging. They felt strongly that the DPP should be designed, packaged, and branded to resemble a contraceptive or a beauty product to minimize associations with oral PrEP and make it easier to conceal from male partners who are not supportive of use. HCPs, noting similar concerns from clients, saw potential for the DPP to alleviate the social and relationship pressures that limit women from taking PrEP as a standalone product; for example, by enabling clients to obtain the DPP at a family planning clinic. One HCP from Kenya stated, “…*if they are taking one pill at the same time, for those who feel they want to hide because they are taking PrEP, it will be easier because they only have one pill to take and so ‘no one should know what I am taking*.’” The DPP was thus widely perceived as a means for women to obtain HIV protection that posed less risk to their existing romantic relationship and their public image in the community and society at large.

#### 3.1.3. Male partners felt the DPP could threaten gender norms, but held more positive individual attitudes

Male partners hold significant influence over SRH decisions and expressed concern that the DPP could threaten gender norms, particularly in rural locations. The majority of male participants said they would not support their own partners to use the DPP. By removing the risk that a woman may become pregnant or acquire HIV, some argued the DPP could enable women to “act like men” and be sexually adventurous with minimal risk of consequences. This in turn was expected to fuel fear and paranoia among men that their female partners might be using the DPP to be unfaithful to them.

Some male romantic partners saw a societal benefit in the DPP, viewing it as an innovation that could help address endemic issues of unintended pregnancy and HIV in their countries and communities. In a number of cases, male partner support for the DPP was caveated with a desire to be involved in DPP rollout, particularly in Kisumu, Kenya. The research indicated that male participants who were younger, urban, highly educated and had a higher socio-economic background would be most likely to support their female partners to use SRH products such as the DPP. Male participants belonging to higher socio-economic backgrounds expressed more progressive views around ensuring their female partner's independence. Urban male participants placed less emphasis on traditional and religious values in their relationships and held more positive individual attitudes towards the DPP. In Zimbabwe, male participants put the most emphasis on presenting as a leader and contributor within their communities. Evidence of positive deviancy highlights an opportunity for the DPP to identify and promote the minority of men who do support DPP uptake and adherence as role models for driving social change and acceptance of the DPP within their communities.

#### 3.1.4. Negative triggers motivate product uptake

Most trigger moments for OCP and oral PrEP uptake were associated with negative emotions, and as such, women strongly identified with the type of person who is prepared to take on risks that are often outside their control. In particular, initiation of oral PrEP was motivated by experiences of untrustworthiness in their male romantic partners, with one Kenyan participant affirming, “*I felt that since he started cheating, I have to protect myself*.” Out of the 13 female oral PrEP users consulted for this research, 11 began using oral PrEP after suspecting or discovering that their partners were unfaithful, and one user initiated oral PrEP upon learning that her partner had kept their positive HIV status secret during their relationship.

In Kenya, partner infidelity prompted some women to start their own affairs as a form of retaliation (“tit for tat”), while in South Africa, some participants cited broader incidences of untrustworthiness, such as male partners cheating on HIV tests or spending their money on extramarital affairs. In Zimbabwe, some oral PrEP/OCP users perceived their relationships to be high-risk. More positive triggers to product entry that participants mentioned included delaying children, for example due to financial burden or focusing on their current child.

#### 3.1.5. DPP user journey and personas

**Figure 2:**
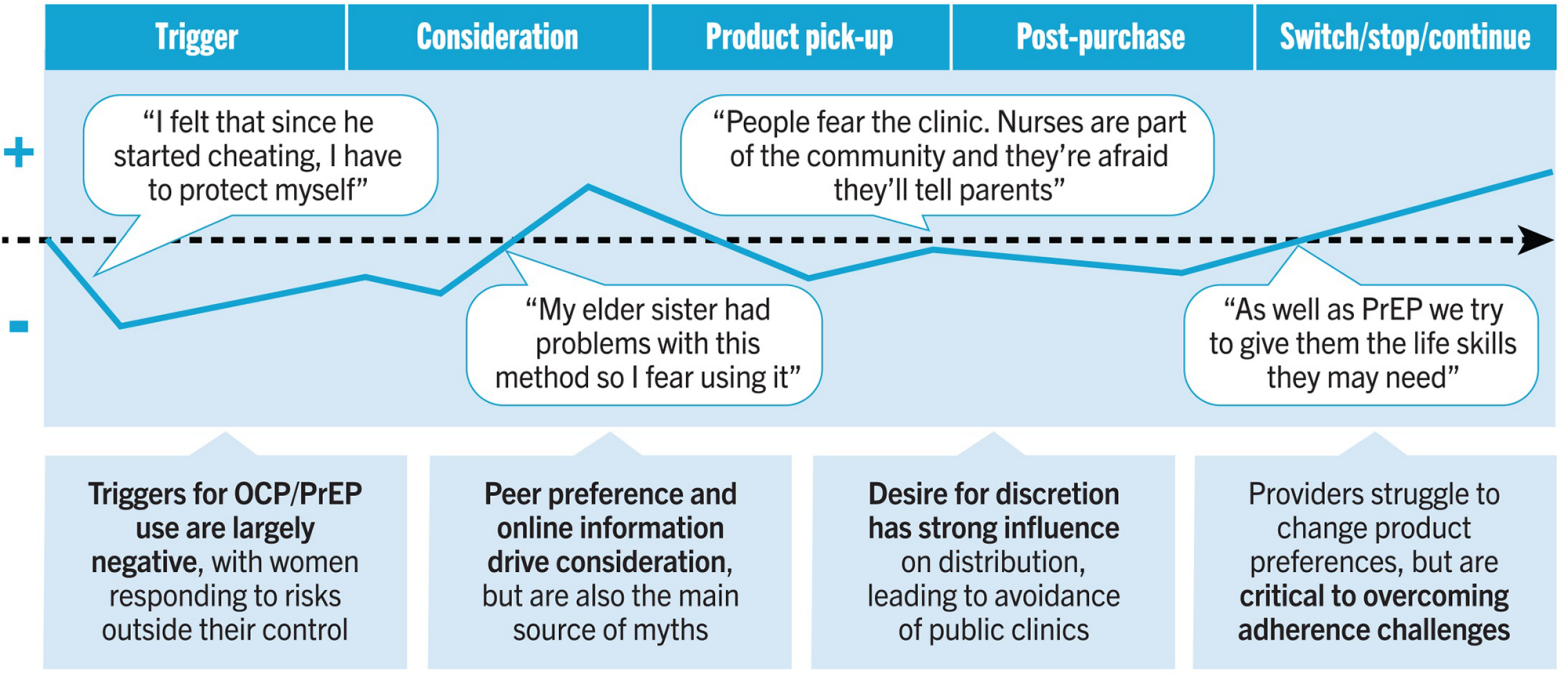
Potential DPP user journey.

The research gathered strong evidence from female participants, HCPs, and cultural commentators indicating that women develop preconceptions and preferences for oral PrEP and OCP well before actually using the products (Figure 2). Female participants described forming a preference based on observing what close family members or friends were using, or because of what they had learned in school. Along the user journey, beliefs were entrenched by peer preference and online information (including social media), which also served as the main source of myths, stigma, and misinformation. Consequently, there was no evidence that female participants changed their minds about a particular product preference if it had been formed before the trigger moment, nor in response to a recommendation by an HCP prior to the moment of initiation. Instead, preferences for a given product tended only to change after the original trigger moment of uptake, such as in response to side effects, and HCPs played a more critical role in supporting adherence.

User journeys demonstrated a general upward trend in the emotional states of the female participants, especially after they had the opportunity to experiment with a product and concluded that it would be suitable for their needs. This decision was often made after side effects had subsided, signaling that the participant had found a product that would be feasibly used in the long term. One participant in Zimbabwe described, “*I started feeling comfortable after using PrEP for a year with me using it and going to get tested over and over again. That is when I concluded that my pills were working.*”

**Figure 3:**
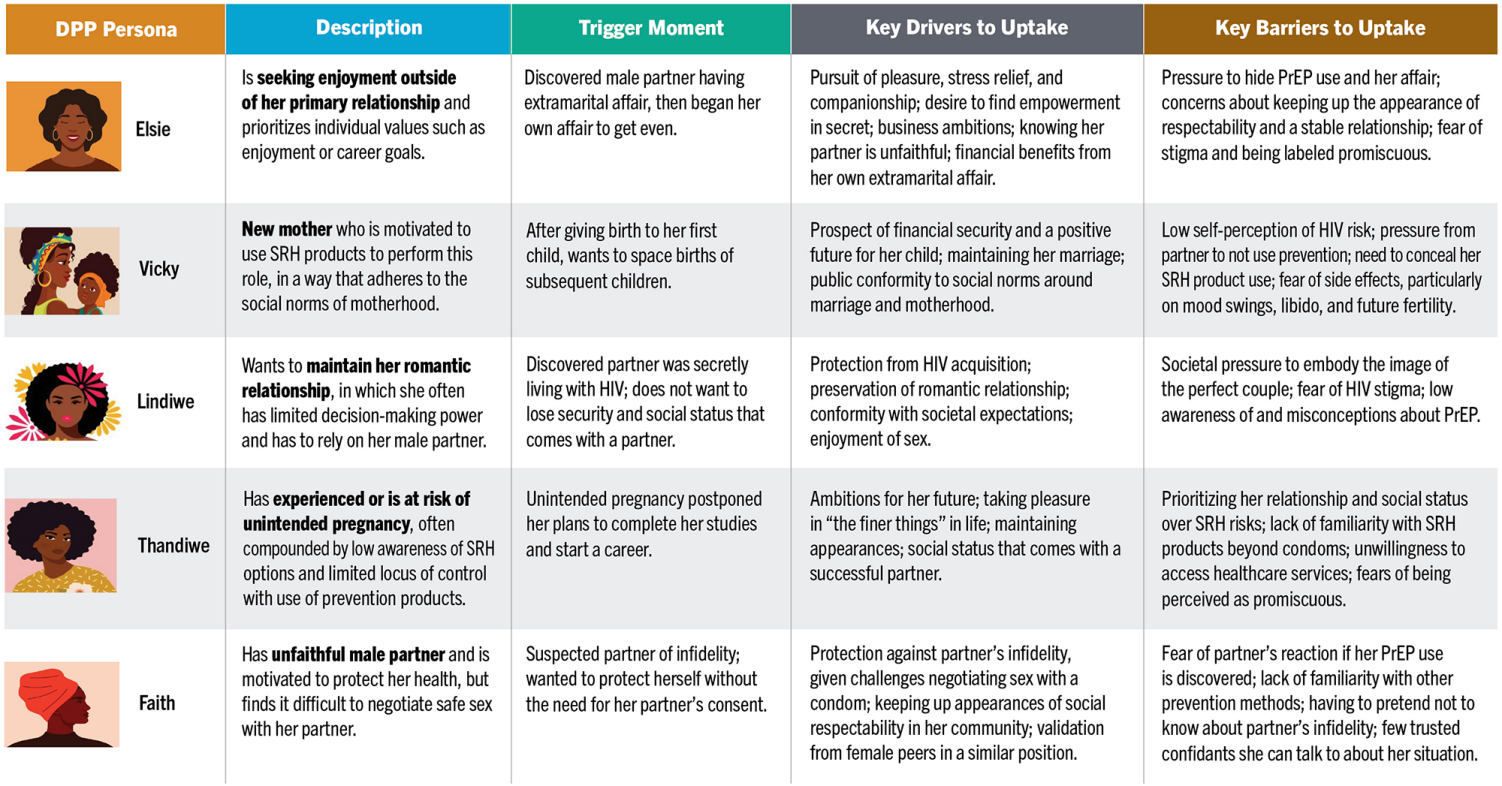
DPP user personas.

These findings demonstrate the need to build DPP awareness and relevance among women prior to trigger moments. Potential triggers for DPP uptake informed the development of five initial DPP audience segments and their barriers and motivations (Figure 3).

Women **seeking enjoyment outside of their primary relationship** demonstrate the highest potential for DPP uptake, prioritizing self-focused values such as enjoyment or career goals. Triggers to PrEP/OCP initiation in this segment are often centered on discovering their male partner was having an extramarital affair and subsequently engaging in their own affair with another man. This persona is concerned about maintaining her reputation as a respectable woman and goes to great lengths to keep her extramarital relationship a secret.

**New mothers** are motivated to use SRH products in a manner that aligns with the social norms of motherhood. This persona likely started using OCP after giving birth to her first child and wants to space the births of subsequent children. A notable barrier is that she does not see HIV prevention as relevant for her; she is also more likely to feel the need to conceal her SRH product use from her partner.

Women who aim to **maintain their romantic relationships** are primarily motivated by relationship goals, which often denotes greater influence of male partners over their decision-making. This persona begins using PrEP upon discovering her partner was living with HIV and does not want to lose the security and social status that comes with having a male partner.

Women who have **experienced or are at risk of unintended pregnancy** face challenges such as limited awareness and locus of control around use of SRH products. This persona began using OCP following an unintended pregnancy, which postponed her plans to complete her studies and start her career. Yet maintaining her relationship was also important to her, as her partner brought her social status and helped fund her lifestyle.

Women whose **male romantic partners are untrustworthy** are motivated to protect their health but struggle to negotiate safe sex with their partner. This persona began using PrEP when she suspected her partner of infidelity, enabling her to protect herself without the need for her partner's consent. She is concerned that a conversation about prevention will upset her partner by implying that she does not trust him.

While the research elucidated these five archetypes as potential DPP users, it is important to note that these personas aggregate many different data and are not based on a single individual. As such, many individuals do not fit into a single persona and can embody different personas or aspects of personas at various moments over the life course. Given this fluidity, the DPP demand generation strategy should be designed to flex across various audiences, leveraging but not always corresponding to the specific personas outlined above.

### 3.2. Creative route for the DPP

**Figure 4:**
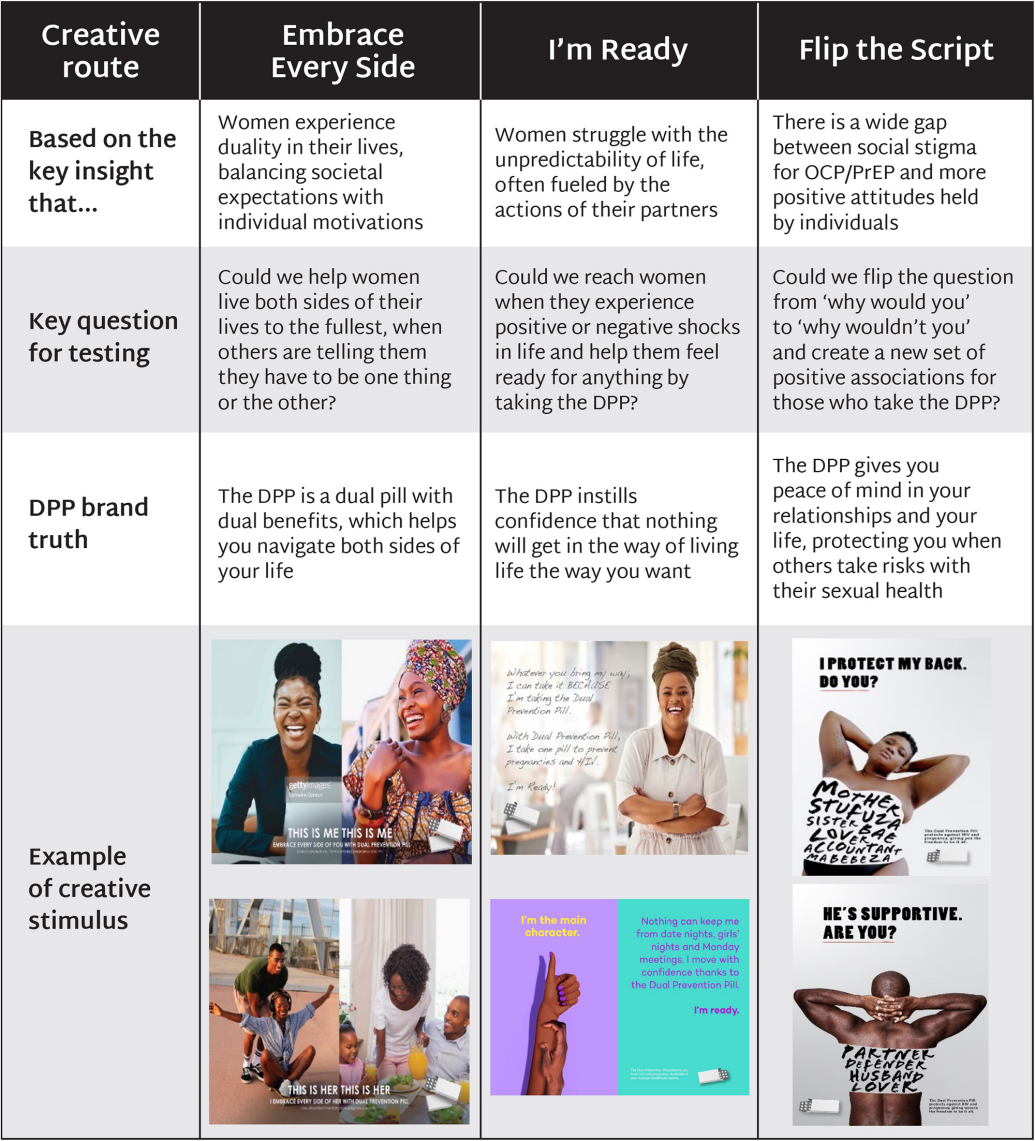
Potential creative routes tested for the DPP in co-creation workshops.

Research findings and user personas underscored the importance of finding a creative route for the DPP that resonated with participants’ broader values and lifestyles and framed the DPP in relation to them, rather than approaching the DPP from a health lens, which occupied a smaller share-of-mind. The findings were used to develop three potential creative routes for the DPP, which were then tested and refined through co-creation workshops with end users, male partners, and HCPs (Figure 4).

#### 3.2.1. Embrace every side

*Embrace Every Side* reflected the insight that women often experience a sense of duality in their lives, needing to balance societal expectations with their own motivations. Creative stimulus for this route depicted different moments of duality in women's lives, such as balancing career aspirations with being a supportive wife and mother; traditional values with wanting a different life than the previous generation; and pleasure and enjoyment with the appearance of respectability. The DPP was positioned as a dual pill with dual benefits that could help women navigate both sides of their lives.

The need to balance multiple sides of their identity resonated with all audiences in co-creation workshops. In particular, career-focused women felt this message captured their everyday reality. Male partners felt that their own experiences balancing multiple identities were overlooked, as they were primarily portrayed as supporters of their female partners.

#### 3.2.2. I’m ready

*I’m Ready* responded to the insight that women struggle with the unpredictability of life, often fueled by the actions of their romantic partners. Creative stimulus featured women who were ready to embrace life, rather than being consumed by the anxieties of SRH. This direction reflected the findings that protection from partners who engaged in risky behavior was more motivating than protection from HIV and a stronger association with women who were “ready for anything” than with women who actively seek to prevent HIV. The DPP was positioned as a product that could instill confidence in women that nothing will get in the way of living the lives they desire.

Participants strongly connected with the need to prepare to weather shocks that are outside of their control. This route performed best when it connected to everyday moments and supported women to achieve career and family goals. Participants expressed concerns that associations with leisure and nightlife could be seen as encouraging promiscuity. Men appreciated being framed as “in it together” with their female partners but were less supportive when the route challenged traditional gender norms.

#### 3.2.3. Flip the script

*Flip the Script* acknowledged the existing social stigma surrounding OCP/PrEP, contrasting it with more positive attitudes held by individuals. Creative stimulus reframed prevention behaviors as a set of positive associations, such as protecting against the actions of others rather than one's own, becoming a desirable partner instead of damaging the relationship, and shifting from the default notion of taking on risks to taking prevention “just in case.” The DPP was positioned as offering peace of mind in women's relationships and lives, protecting them when others take risks with their sexual health.

Participants agreed that stigma around OCP/PrEP needed to be changed but expressed concerns that the selected imagery, which showed too much skin, could reinforce perceptions of promiscuity. However, participants resonated with written affirmations on the images, which they believed could help reduce stigma.

#### 3.2.4. Selected creative route

**Figure 5:**
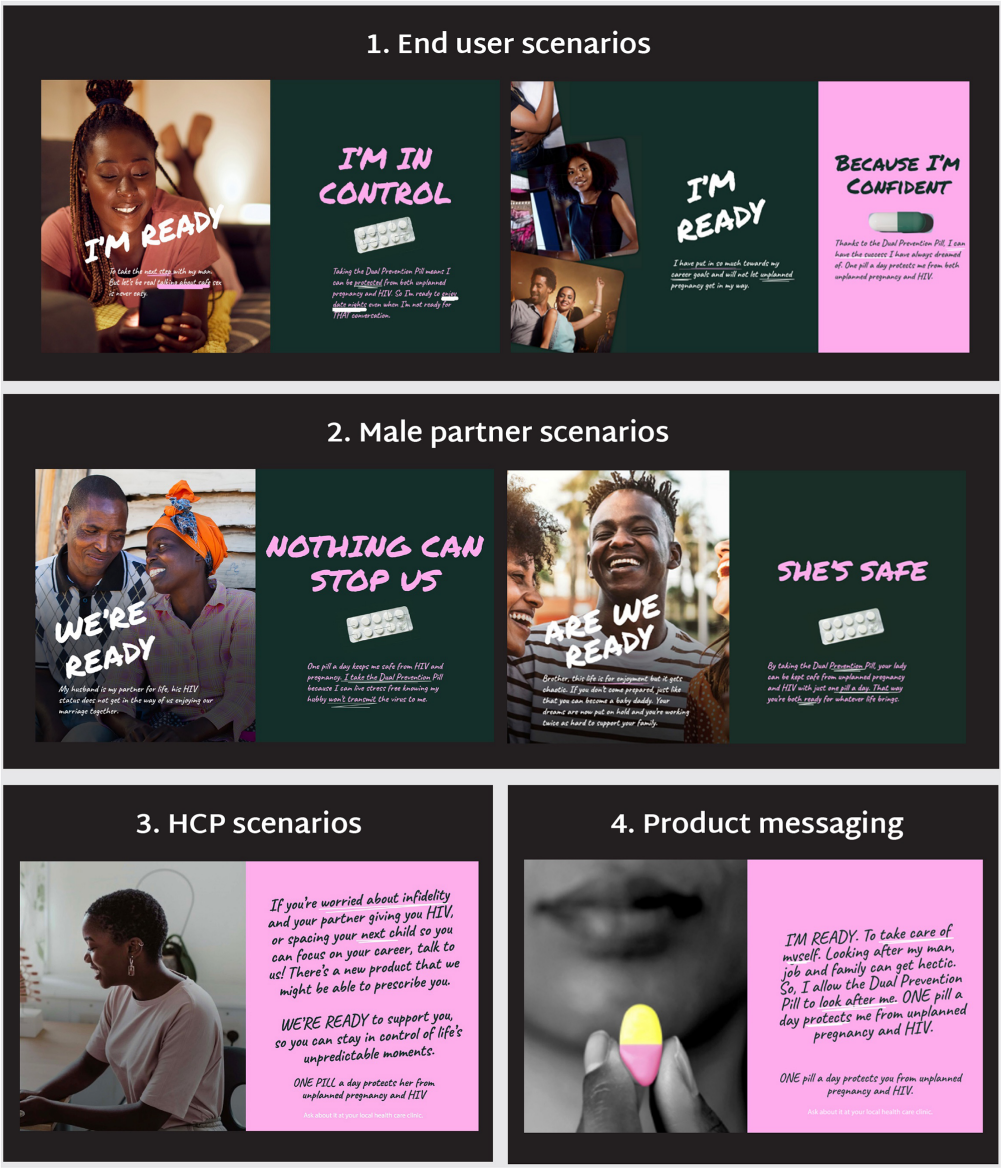
Examples of creative stimulus tested in validation workshops.

After comprehensive testing*, I’m Ready* was found to be the preferred route across all markets and audiences. Creative stimulus was refined, expanded and tested in validation workshops with end users, male partners, and HCPs to understand if participants understood, liked, and connected with the creative concept, scenarios and messaging (Figure 5).

Comprehension of the scenarios and product benefits was consistently high across all audiences and geographies. End users underscored the need to avoid ambiguity in messaging, as headlines with a question-and-answer format (e.g., “Am I Ready? Yes”) and messaging intended to be more discreet led to confusion. Participants related to most, if not all, of the scenarios and agreed that most executions would be appropriate in public spaces to increase reach and awareness. Male partners felt making the messaging accessible would help protect the women in their lives and foster conversations within friend groups and relationships. Scenarios addressing experiences of infidelity deeply resonated across groups, but participants preferred softer language to communicate this, as a female participant from Zimbabwe noted: “*We may not like how blunt the message is, but this is real- it's the reality.”*

Potential end users felt motivated by stimulus that celebrated their individuality as a woman, whether through a goal-oriented or self-care lens. Women appreciated a focus on how pregnancy and HIV impact their lives, and how protecting themselves benefits their current well-being and long-term goals. Both women who already prioritized self-focused values as well as those who felt they needed encouragement to do so responded positively to these messages.

In addition, end users appreciated references to intimacy, particularly where partners were depicted enjoying their sex lives regardless of HIV status, and they appreciated a broad range of messaging for diverse populations regardless of HIV status, life stage, and gender. End users requested more inclusive language to expand the applicability of enjoyment and pleasure scenarios, such as changing “husband” to “partner” or “campus” to a more general setting. Male partners were less supportive of inclusive messaging and responded negatively to scenarios featuring sero-discordant couples.

The stimulus made male partners appreciate the need for the DPP. Male partners across all countries most responded to messaging that positioned them as protectors of women in their lives who were not their romantic partners, recognizing that it was more difficult for them to discuss SRH with their wives. However, they felt positively about advising female friends and relatives to use the DPP and appreciated that these messages could help break down socio-cultural barriers around men speaking with female friends and relatives about sexual health.

Male partners also responded positively and enthusiastically to messages that focused on enjoyment, which appealed to their desire to not be affected by the consequences of their lifestyles. This message ranked highly among those they wished to see across public spaces because they felt it could prompt conversations in their friend groups. One male participant in South Africa affirmed, “*Everyone of us can relate to this scenario and we do want the best for the women in our lives*.”

HCPs preferred creative executions that focused on promoting empowerment and control, rather than narratives framing women as victims of infidelity by their male partners. HCPs found messages that positioned them as supportive of the priorities of their clients, such as spacing children to focus on their careers, would be powerful because they were both needed and relatable. HCPs underscored that messaging that made their clients feel confident in their choices would resonate, and requested additional information to support clients with side effects, continuation, and switching to this end.

Building on the primary research finding that HCPs wielded greater influence later in the user journey, HCPs were shown a range of adherence tools to understand which they felt would be useful to support clients to use the DPP. A daily reminder app to encourage adherence was highlighted as a particularly effective measure that could reach a large number of women and allow for discreet tracking. Discreet packaging was also preferred, potentially with a visual or mechanism to encourage adherence.

## 4. Discussion

Taken together, findings from this research provide a strong creative foundation for designing a comprehensive demand generation strategy for the DPP. Four key recommendations surfaced for generating demand for the DPP, which are elaborated on in this section.

**Connect with women's diverse identities and aspirations:** The research findings point to an opportunity for highly distinctive communications on the DPP that reflect the interplay of different values, rather than focusing on a singular value, which risks diluting the complex lived realities and multiple goals of end users. Focusing on women in their diversities aligns with a notable shift in the broader SRH field away from risk-based communications and towards communications that elevate values, goals, and needs that may appeal to end users, particularly around self-care and women's empowerment (19, 20). For instance, the Kotex “She Can” campaign in Kenya demonstrates the importance of balancing aspirational messages with people's lived reality, as it was criticized for being overly positive (e.g., women can do anything on their periods) and downplaying the realities and challenges of having a period (e.g., pain, social stigma) (16). There is an opportunity to position the DPP as a product that makes it easier for women to juggle competing values without having to make trade-offs for their SRH, and could even enhance their enjoyment and intimacy. Finding the right balance between reality and aspiration will be critical for DPP communications.

**Link the DPP with relationship goals:** The research found that OCP and PrEP are viewed as products that can put people's romantic relationships at risk, and that women value maintaining their relationships above SRH goals. Other studies across sub-Saharan Africa have similarly found that relationship goals are more relevant than HIV prevention for women, and in particular AGYW, and they will prioritize the needs and preferences of their romantic partners over their own (21, 22).

Discussion of male partners as barriers to women's PrEP and contraceptive use is widespread in the literature. Examples include anticipated and actual accounts of behaviors that limit a woman's capability, opportunity, and motivation to initiate and adhere to prevention products, including contraceptive sabotage, intimate partner violence (IPV), and threats of divorce (23–26). In particular, literature highlights women's concerns that discovery or disclosure of PrEP or contraceptive use will be interpreted by their partner as distrust or implications of unfaithfulness (25), and has found discretion to be a highly valued product attribute (27). Several studies highlight the importance of facilitating covert access to and use of contraception and PrEP as strategies for avoiding stigma while remaining protected from unintended pregnancy and/or HIV (23, 24, 27). Moreover, studies suggest that a lack of communication about prevention use can decrease adherence among women who try to hide their use of products, and conversely, that disclosure, when successful, can increase adherence (27–29).

Some male participants recruited for this research may have been positive deviants with respect to the dominant social norms and practices of their communities, as the positivity of their responses to the DPP was in contradiction to what the literature dictates. Yet romantic relationships are not monolithic, and some are more conducive to women's use of prevention products. A recent qualitative study in South Africa identified four distinct relationship typologies, each with different likelihoods of adoption of HIV self-testing (25). Several studies highlight the heterogeneity of male attitudinal and behavioral profiles related to HIV prevention (30). Literature also indicates that, when supportive, male partners can play an invaluable role in encouraging their partners to use and adhere to products like the DPP, for example by creating an accepting environment and reminding them to take their daily dose (27, 29, 31). Perspectives are mixed on the extent to which men should be involved in women's SRH decision-making as a trade-off to potentially increasing acceptability (32), and DPP messaging should be careful not to reinforce existing stigma around women's agency to make their own decisions on SRH.

Previous HCD research on the DPP found that women do not want to have to be discreet with their product use, and a saw a role for mass media campaigns to combat societal stigma around prevention (33). This research revealed a consensus view among HCPs and cultural commentators that men needed to be proactively targeted to support the DPP in order to limit the risk of product stigmatization among men. Placing DPP campaigns in public spaces were found to be a strong potential entry point to transform male attitudes towards prevention in the broader community, especially when messaging positioned men's support for the DPP as a way to protect women in their lives, given that men were largely resistant to the DPP in the research. Men felt scenarios depicting their own enjoyment could catalyze conversations on sexual health with their peers, indicating that DPP campaigns should seek to reach men with potential for positive deviancy who could influence their peer networks. In addition, there is an opportunity to flip the perception that DPP use could put relationships at risk by showing that DPP users care about their partner and protecting their relationship. At the same time, communications can reframe the DPP as for women who are savvy about protecting themselves from the actions of others, thereby shifting stigma away from end users.

**Leverage different triggers for media targeting:** Most triggers to OCP and PrEP use relate to negative experiences, such as entry into an established risk group, including commercial sex workers, people with multiple sexual partners and sero-discordant couples, discovery or suspicion of a partner's sexual behavior outside of the relationship, and negative experiences with other prevention methods (29, 31, 34). Literature about how influencers in a woman's social network interpret prevention use tend to focus on negative trigger moments exclusively. Negative trigger moments are thus likely to be significant category entry points for the DPP that can be targeted through media placement. Relevant positive trigger moments, such as change in relationship status, starting university, or child spacing, can be leveraged too (25, 29, 35). Other point-in-time segmentations demonstrate how AGYW may transition in and out of need-states when they are more likely to use a product like the DPP (36, 37). There is an opportunity to position the DPP as helping women to overcome moments of uncertainty and navigate moments when they feel they need to take control of their sexual health, as well as to broaden the concept of “readiness” to apply to positive triggers for adoption, such as preparing for university, a night out, or marriage.

**Strike a balance in channel placement:** Research findings pointed to high potential for a multifaceted demand generation strategy for the DPP that simultaneously raises public awareness to create social acceptability and delivers more targeted, discreet communications to end users in safe and trusted channels for messaging that male partners and communities may view as controversial. In Kenya, the Jilinde project's “360-degree” marketing campaign targeting multiple levels to effectively shape perceptions of PrEP country-wide, utilizing national mass media and local events to create widespread awareness and position PrEP as a product for anyone at risk of HIV helped to mitigate potential stigma (36). These campaigns were followed by a large increase in oral PrEP uptake in some counties. Other mass media campaigns have also been effective at creating acceptability and increasing uptake (38). More targeted efforts focused on reaching specific audiences have also been found to drive uptake and support amongst those groups, such as peer-to-peer approaches and men's engagement (36, 39).

## 5. Conclusion

This research provided valuable insights into the barriers and motivators for DPP uptake, which can be leveraged to develop demand generation strategies for the DPP. The use of HCD techniques ensured a deep understanding of participants’ perspectives and cultural contexts, contributing to the relevance and effectiveness of the research findings. Moreover, research findings are applicable to MPTs as a product category, as they draw from both HIV prevention and family planning as well as on broader relationship and SRH dynamics. With a myriad of MPTs in the research pipeline, lessons from demand generation for the DPP can be leveraged to inform future MPT introduction, enabling prevention products and services to reach more end users through their preferred channels and approaches.

## Data Availability

The raw data supporting the conclusions of this article will be made available by the authors, without undue reservation.
