# Corrigendum: How might we motivate uptake of the Dual Prevention Pill? Findings from human-centered design research with potential end users, male partners, and healthcare providers

**DOI:** 10.3389/frph.2023.1341771

**Published:** 2023-12-05

**Authors:** Wawira Nyagah, Kate Segal, Jess Feltham, Alex Ash, Jocelyn Major, Moowa Masani

**Affiliations:** ^1^ AVAC, Product Introduction and Access, New York, NY, United States; ^2^ M&C Saatchi World Services, London, United Kingdom; ^3^ REACH Consumer Insights, Cape Town, South Africa

**Keywords:** pre-exposure prophylaxis, oral contraception, HIV prevention, family planning, multi-purpose prevention technologies, end users, human-centered design, demand generation

A Corrigendum on How might we motivate uptake of the Dual Prevention Pill? Findings from human-centered design research with potential end users, male partners, and healthcare providers By Nyagah W, Segal K, Feltham J, Ash A, Major J, Masani M, (2023). Front. Reprod. Health 5:1254953. doi: 10.3389/frph.2023.1254953

**Text correction**

In the published article, one of our contributors was omitted from the Acknowledgements section of the article. A correction has been made to **Acknowledgements**. This section previously stated:

“The authors are grateful for the generous support from the Children's Investment Fund Foundation (CIFF) for this research. The contents of this paper are the sole responsibility of the authors and do not necessarily reflect the views of CIFF. The authors would like to thank colleagues at AVAC and M&C Saatchi World Services for their guidance on the research and review of the manuscript, and members of the DPP Advisory Board and DPP Civil Society Advisory Group for providing feedback on the research at multiple stages throughout the process. Finally, the research would not have been possible without the critical contributions of field research teams and research participants in Kenya, South Africa, and Zimbabwe.”

The corrected section appears below:

“The authors are grateful for the generous support from the Children's Investment Fund Foundation (CIFF) for this research. The contents of this paper are the sole responsibility of the authors and do not necessarily reflect the views of CIFF. The authors would like to thank colleagues at AVAC and M&C Saatchi World Services for their guidance on the research and review of the manuscript. Humanly supported the design of HCD research tools, trained field research teams, analyzed outputs of the HCD research, and helped develop the user journeys and personas. Members of the DPP Advisory Board and DPP Civil Society Advisory Group provided feedback on the research at multiple stages throughout the process. Finally, the research would not have been possible without the critical contributions of field research teams and research participants in Kenya, South Africa, and Zimbabwe.”

The authors apologize for this error and affirm that this does not change the scientific conclusions of the article in any way. The original article has been updated.

